# Spino cranial angle as a predictor of loss of cervical lordosis after laminoplasty in patients with cervical myelopathy

**DOI:** 10.1186/s12893-021-01293-1

**Published:** 2021-06-12

**Authors:** Zheng Wang, Jia-Xin Xu, Zhen Liu, Ruo-Yu Li, Zhi-Wei Wang, Heng-Rui Chang, Wen-Yuan Ding, Da-Long Yang

**Affiliations:** Department of Spinal Surgery, The Third Hospital of Hebei Medical University, 139 Ziqiang Road, Shijiazhuang, 050051 People’s Republic of China

**Keywords:** Spino cranial angle, Loss of cervical lordosis, Laminoplasty, Cervical alignment

## Abstract

**Background:**

To explore the relationship between spino cranial angle (SCA) and loss of cervical lordosis (LOCL), and to determine whether SCA has the ability to predict LOCL for patients with cervical myelopathy.

**Methods:**

A total of 68 consecutive patients with cervical myelopathy who received laminoplasty (LAMP) were selected to the current study. C2–C7 lordosis was defined as a representation of the cervical alignment. Alignment change > 0° was considered LOCL. Multiple linear regression analysis was applied to evaluate the association between LOCL and various sagittal parameters at preoperative, such as SCA, CL, T1s and cSVA. Linear regression analysis was applied to evaluate the relationships between LOCL and preoperative SCA in each subgroup.

**Results:**

Patients were assigned to three groups depending on the quartile of preoperative SCA. The first quarter of patients were defined as the low SCA group, the last quarter were defined as the high SCA group and the middle half were defined as the middle SCA group. There was no statistically significant difference in age, sex and the type of OPLL among the three groups. Patients in the low SCA group showed more cervical lordosis before surgery and more LOCL after LAMP (*p* < 0.001). After linear regression analysis for SCA and LOCL, preoperative SCA was negatively correlated with LOCL in the low SCA group (*r* = − 0.857, *p* < 0.001) and high SCA group (*r* = − 0.515, *p* = 0.034). However, there was no significant correlation between preoperative SCA and LOCL in the middle SCA group (*r* = 0.027, *p* = 0.881).

**Conclusions:**

Patients with lower SCA had more lordosis preoperatively and performed more LOCL after LAMP at 2 years of follow-up. Both too high or low preoperative SCA were negatively correlated with the degree of LOCL, while when the SCA fluctuates in a suitable range, it is easier to compensate for the changes of cervical sagittal alignment.

## Background

Laminoplasty (LAMP) is commonly used in patients with cervical spondylotic myelopathy (CSM), developmental and degenerative cervical spinal stenosis, and ossification of posterior longitudinal ligament (OPLL), which is an effective posterior approach. It preserves the range of motion of cervical spine and does not cause instability [1]. The incidence of kyphosis after LAMP is relatively low [2]. However, as a kind of posterior surgery, preoperative and postoperative curvature maintenance is the premise of success. It is imperative to restore the physiological curvature of cervical spine for maintaining the long-term stability of cervical spine and restoring the biomechanical environment of cervical spine itself. The loss of physiological lordosis leads to the decrease of spinal canal volume, and the increase of kyphosis leads to the increase of spinal cord tension, which leads to poor postoperative outcomes. Although patients undergoing LAMP have enough lordosis before operation [3], there are often changes in sagittal alignment after operation. However, this change in the sagittal balance after LAMP may reduce the surgical outcome and require additional operation [4]. Though cervical sagittal alignment changes have been found to perform a close correlation with the quality of life [5, 6], there are few researches on the preoperative risk factors of sagittal alignment changes after LAMP. As a predictor of loss of cervical lordosis (LOCL), T1 slope (T1s) has been widely concerned and recognized [7–11]. So we will focus on another equally important sagittal parameter: spino cranial angle (SCA), which is obtained between a line drawn from the centre of the sella turcica and a tangent to the upper C7 plateau [12] and has been reported to present a significant correlation with many sagittal parameters [13]. It is the first parameter to connect the base of cervical spine with the weight of head and creatively put forward the concept of head offset, which is supposed to be the focus of future research [14].

The aims of this study were to explore the relationship of SCA and LOCL after LAMP for cervical myelopathy, and to identify whether SCA could be used as a predictor of LOCL.

## Methods

### Patient population

We retrospectively reviewed 68 consecutive patients with CSM or OPLL who underwent LAMP with a plate fixation system between January 2014 and December 2018 and divided them into three categories depending on the quartile of preoperative SCA. The first quarter of patients were defined as the low SCA (LS) group, the last quarter were defined as the high SCA (HS) group and the middle half were defined as the middle SCA (MS) group. We included patients with (1) CSM or OPLL are diagnosed in the clinic, computed tomography (CT) and magnetic resonance imaging (MRI); (2) follow-up for not less than two years. We excluded patients with: (1) history of previous cervical surgery; (2) cervical trauma, tumors, or infections; (3) sagittal alignment parameters were too difficult to measure. All the surgeries were finished successfully using a plate fixation system and the interspinous ligaments were preserved during surgery. For all C2 level, dome osteotomy was achieved but complete osteotomy of C2 spinous process was not executed. All patients were told to wear a Philadelphia neck collar for 4 weeks at postoperative.

### Radiographic analysis

**Fig. 1:**
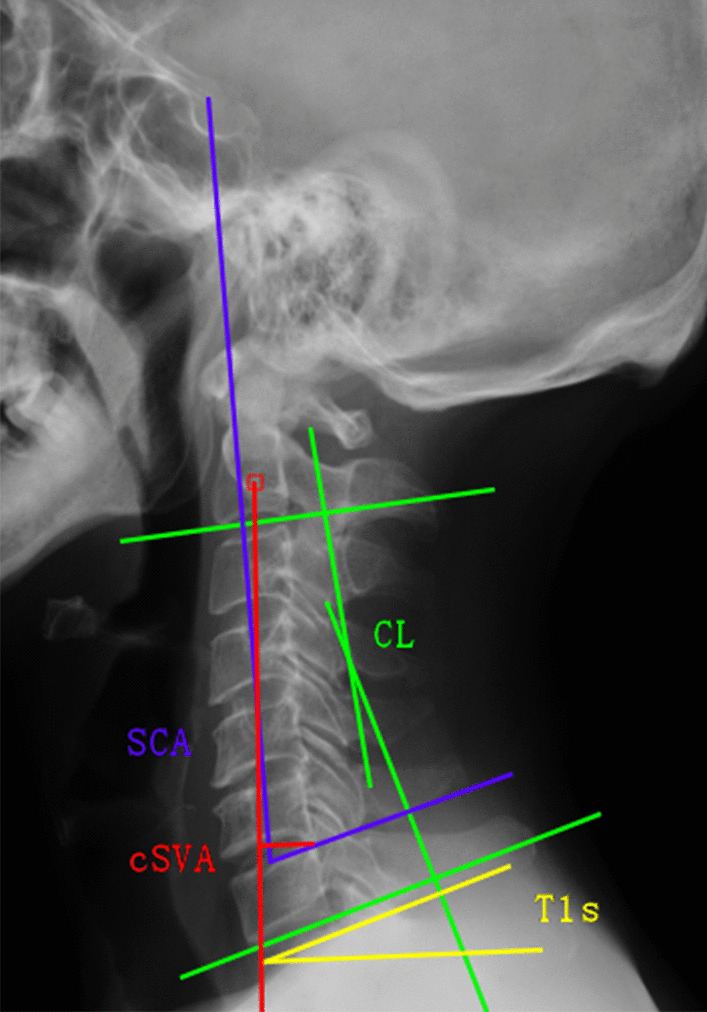
Spino cranial angle (SCA): the angle is defined between the C7 slope and the straight line joining the middle of the C7 endplate and the middle of the sella turcica; T1 slope (T1s): the angle between a horizontal line and the superior endplate of T1; C2–C7 lordosis (CL): the angle created by a line parallel to the inferior aspect of the C2 body and a line parallel to that of the C7 body; C2–C7 sagittal vertical axis (cSVA): horizontal distance between the center of C2 and the posterior edge of the upper endplate of C7

Lateral radiographs were taken when the patient was in a comfortable standing position, with the upper extremities attached naturally at the side of the trunk and while facing forward. Radiological parameters included SCA, T1s, C2-C7 lordosis (CL) and C2-C7 sagittal vertical axis (cSVA), which were measured (Fig. 1): (1) SCA is defined as the angle defined between a line drawn from the centre of the sella turcica and a tangent to the upper C7 plateau. (2) T1s is defined as the angle between a horizontal line and the T1 superior endplate. (3) CL is defined as the angle formed by the inferior endplates of C2 and C7, which was considered to be the measurement of cervical alignment. Lordosis was presented as a positive angulation, and kyphosis as negative [8, 10, 15, 16]. Changes in the cervical alignment were assessed by the following [10, 11, 15]: alignment changes (°) = (preoperative CL) − (postoperative CL). Based on the formula, alignment changes > 0° were regarded as LOCL. According to the description of alignment change, the incidence of LOCL over 5° and 10° was also evaluated. (4) cSVA is obtained by dropping a vertical line from the center of the C2 body and measuring the distance in millimeters between this plumb line and the posterior superior corner of C7. All lateral measurements were collected before surgery and at latest follow-up.

### Statistical analysis

All data were statistically calculated by SPSS (version 22.0; SPSS Inc., Chicago, IL, USA). Measurement data are expressed as mean ± standard deviation. Each independent variable was compared among the three groups using the independent-sample t-test or Mann–Whitney U, and the Pearson Chi-square test, Fisher Exact or Linear-by-linear association test. Multiple linear regression analysis was applied to evaluate the relationship between LOCL and preoperative various sagittal parameters, such as SCA, T1S, CL and cSVA. Linear regression analysis was applied to verify the correlations between LOCL and preoperative SCA in each subgroup. *p* values of < 0.05 were considered statistically significant.

## Results

### Comparison of patient backgrounds depending on preoperative SCA

Patient clinical features depending on preoperative SCA are summarized in Table 1. The value of SCA varied from 69.3° to 75.9° in the LS group, from 76.2° to 92.3° in the MS group and from 93.4° to 104.9° in the HS group. Age, sex and type of OPLL failed to reach significance among the three groups.

**Table 1:** Comparison of patient backgrounds

	Total	LS	MS	HS
No. of patients	68	18	33	17
Age (year)	58.63 ± 8.67	57.39 ± 9.44	59.39 ± 7.73	58.47 ± 9.89
Sex (M/F)	35/33	11/7	16/17	8/9
Range of SCA (º)	69.3–104.9	69.3–75.9	76.2–92.3	93.4–104.9
CSM/OPLL	45/23	13/5	23/10	9/8
Type of OPLL (N)				
Segmental	12	2	5	5
Continuous	5	1	2	2
Mixed	6	2	3	1

	LS vs. MS	LS vs. HS	MS vs. HS
Age (year)	0.417^*^	0.447^#^	0.926^#^
Sex (M/F)	0.388^+^	0.505^†^	0.924^+^
CSM/OPLL	0.850^+^	0.305^†^	0.242^+^
Type of OPLL (N)	0.779^‡^	0.347^‡^	0.578^‡^

*SCA* spino cranial angle, *CSM* cervical spondylotic myelopathy, *OPLL* ossification of posterior longitudinal ligament, *LS* low SCA, *MS* middle SCA, *HS* high SCA

*Independent t-test

^#^Mann–Whitney U test

^+^Pearson Chi-square test

^†^Fisher’s Exact test

^‡^Linear-by-linear association

### Comparison of sagittal parameters depending on preoperative SCA

The values and differences of sagittal parameters among the three groups are summarized in Table 2. Regardless of preoperative or postoperative, only T1s revealed no significant difference between the MS and HS group. Besides, all other included indicators demonstrated significant differences among the three groups. Patients in the LS group often had higher T1s, CL, and lower cSVA, while patients in the HS group showed lower T1s, CL, and higher cSVA. The mean values of sagittal parameters in the MS group were between LS and HS group. None of the patients we included showed kyphosis cervical alignment before surgery. Postoperative kyphosis occurred 11.1% (2/18) of patients in the LS group, 11.8% (2/17) of patients in the HS group and 3.0% (1/33) of patients in the MS group. However, there is no significant statistical difference among the three groups.

**Table 2:** Comparison of preoperative and postoperative sagittal parameters depending on the preoperative SCA

	Total	LS	MS	HS
Pre-SCA (°)	84.08 ± 9.68	72.52 ± 2.21	83.73 ± 4.84	97.01 ± 3.50
Post-SCA (°)	88.22 ± 9.64	78.66 ± 8.72	88.61 ± 6.41	97.58 ± 5.50
Pre-T1s (°)	25.74 ± 6.26	33.23 ± 4.32	23.78 ± 4.63	21.61 ± 3.51
Post-T1s (°)	23.94 ± 5.85	29.12 ± 4.73	22.20 ± 4.85	21.83 ± 5.59
Pre-CL (°)	15.20 ± 6.27	22.87 ± 3.21	14.25 ± 2.72	8.94 ± 5.37
Post-CL (°)	10.76 ± 5.90	14.73 ± 7.13	11.85 ± 4.15	6.95 ± 4.64
Pre-cSVA (mm)	24.66 ± 9.08	14.17 ± 4.54	25.11 ± 5.76	34.89 ± 4.66
Post-cSVA (mm)	28.66 ± 9.17	22.09 ± 5.75	27.92 ± 8.88	37.05 ± 5.88
Incidence of pre-kyphosis	0%	0%	0%	0%
Incidence of post-kyphosis	7.4%(5/68)	11.1% (2/18)	3.0% (1/33)	11.8% (2/17)

	LS vs. MS	LS vs. HS	MS vs. HS
Pre-SCA (°)	< 0.001^#^	< 0.001^#^	< 0.001^#^
Post-SCA (°)	< 0.001^#^	< 0.001^#^	< 0.001^#^
Pre-T1s (°)	< 0.001^#^	< 0.001^#^	0.137^#^
Post-T1s (°)	< 0.001^*^	< 0.001^*^	0.809^*^
Pre-CL (°)	< 0.001^*^	< 0.001^#^	< 0.001^#^
Post-CL (°)	< 0.001^#^	< 0.001^#^	< 0.001^#^
Pre-cSVA (mm)	< 0.001^#^	< 0.001^#^	< 0.001^#^
Post-cSVA (mm)	< 0.001^#^	< 0.001^#^	< 0.001^#^
Incidence of postoperative kyphosis	0.280^†^	1.000^†^	0.264^†^

*SCA* spino cranial angle, *T1s* T1-slope, *CL* C2–7 lordosis angle, *cSVA* C2–7 sagittal vertical axis, *LS* low SCA, *MS* middle SCA, *HS* HIGH SCA

*Independent t-test

^#^Mann–Whitney U test

^†^Fisher’s Exact test

### Comparison of sagittal alignment changes depending on preoperative SCA

Table 3 summarizes the changes in postoperative alignment. The mean values of changes of T1s were − 4.11° in the LS group, − 1.58° in the MS group and 0.22° in the HS group, respectively. Only the LS group and the HS group showed significant differences (*p* = 0.021). The mean values of postoperative LOCL were 8.09° in the LS group, 2.39° in the MS group and 2.00° in the HS group, respectively. Patients in the LS group performed significantly more LOCL (8.09° vs. 2.39° in the MS group, *p* < 0.001; 8.09° vs. 2.00° in the HS group, *p* = 0.001) and more changes in cSVA (7.92 mm vs. 2.82 mm in the MS group, *p* = 0.003; 7.92 mm vs. 2.16 mm in the HS group, *p* = 0.006). Meanwhile, the occurrence of LOCL > 5° and LOCL > 10° in the LS group significantly exceeded that in the MS group (LOCL > 5°: *p* = 0.006; LOCL > 10°: *p* = 0.047). However, neither the LS group nor the MS group showed significant differences compared with the HS group.

**Table 3:** Comparison of sagittal alignment changes depending on preoperative SCA

	Total	LS	MS	HS
Changes in SCA (°)	4.14 ± 6.49	6.13 ± 8.73	4.88 ± 5.02	0.57 ± 5.12
Changes in T1s (°)	− 1.80 ± 4.93	− 4.11 ± 5.23	− 1.58 ± 4.12	0.22 ± 5.33
Changes in cSVA (mm)	4.01 ± 6.54	7.92 ± 4.92	2.82 ± 6.47	2.16 ± 6.77
LOCL (°)	4.42 ± 3.96	8.09 ± 5.93	2.39 ± 3.20	2.00 ± 3.03
LOCL > 5° (N)	27.9%(19/68)	61.1%(11/18)	15.2%(5/33)	17.6%(3/17)
LOCL > 10° (N)	8.8%(6/68)	27.8%(5/18)	3.0%(1/33)	0%(0/17)

	LS vs. MS	LS vs. HS	MS vs. HS
Changes in T1s (°)	0.064^*^	0.021^*^	0.193^*^
Changes in cSVA (mm)	0.003^#^	0.006^#^	0.894^#^
LOCL (°)	< 0.001^#^	0.001^#^	0.894^*^
LOCL > 5° (N)	0.006^+^	0.086^†^	0.941^‡^
LOCL > 10° (N)	0.047^†^	0.338^†^	1.000^†^

*SCA* spino cranial angle, *T1s* T1-slope, *cSVA* C2–7 sagittal vertical axis, *LOCL* loss of cervical lordosis, *LS* low SCA, *MS* middle SCA, *HS* high SCA

*Independent t-test

^#^Mann–Whitney U test

^+^Pearson Chi-square test

^†^Fisher’s Exact test

^‡^Continuity Correction test

### Multiple linear regression analysis of the relationship between LOCL and sagittal parameters

The results of multiple linear regression analysis are summarized in Table 4. We have defined the postoperative sagittal alignment change as LOCL > 0° in the previous description and included Pre-SCA, Pre-T1s, Pre-CL and Pre-cSVA as factors affecting LOCL. Unfortunately, only T1s was associated with LOCL (Pre-T1s: *p* = 0.003), other selected variables above showed no significant correlation with LOCL in multiple linear regression analysis (Pre-SCA: *p* = 0.376; Pre-CL: *p* = 0.247; Pre-cSVA: *p* = 0.307).

**Table 4:** Multiple linear regression analysis of the relationship between LOCL and preoperative measurements

Variable	LOCL	
	Unstandardized coefficient (B)	*p*
Pre-SCA	0.128	0.376
Pre-T1s	0.367	0.003*
Pre-CL	0.156	0.247
Pre-cSVA	− 0.129	0.307

*SCA* spino cranial angle, *T1s* T1-slope, *CL* C2–7 lordosis angle, *cSVA* C2–7 sagittal vertical axis, *LOCL* loss of cervical lordosis

Dependent variable = LOCL. *p < 0.01

### Linear regression analysis for SCA and LOCL in SCA subgroups

We evaluated the univariate linear regression analysis between SCA and LOCL in the LS, MS and HS group respectively (Table 5). Preoperative SCA was negatively correlated with LOCL in the LS group (*r* = − 0.857, *p* < 0.001) and HS group (*r* = − 0.515, *p* = 0.034). However, there was no significant correlation between preoperative SCA and LOCL in the MS group (*r* = 0.027, *p* = 0.881).

**Table 5:** Linear regression analysis for preoperative SCA and LOCL in SCA subgroups

		LOCL
LS	*r*	− 0.857
	*p*	< 0.001^*^
MS	*r*	0.027
	*p*	0.881
HS	*r*	− 0.515
	*p*	0.034^†^

*SCA* spino cranial angle, *T1s* T1-slope, *CL* C2–7 lordosis angle, *cSVA* C2–7 sagittal vertical axis, *LOCL* loss of cervical lordosis

^†^Correlation is significant at the 0.05 level (two-tailed)

*Correlation is significant at the 0.01 level (two-tailed)

## Discussion

Recently, the significance of cervical alignment balance based on sagittal parameters has been gradually generalized [8, 17]. Sagittal malalignment has been confirmed to be closely associated with a decline in health status, and rational balanced state could be contributing to keep posture and alleviate the quality of life [18–20]. SCA, as the first sagittal parameter to connect the foundation of cervical spine with the weight of head, has been reported the usefulness in evaluating sagittal balance [14], which fluctuates within a certain range (83° ± 9°) under normal conditions and is significantly correlated with T1s and CL [13]. Although the essential sagittal parameter of SCA is gradually familiar, there are limited reports on the role of SCA in sagittal balance [12–14, 21, 22], and whether SCA has the ability to predict the changes of sagittal alignment and clinical results like T1s is still unclear [7, 11]. Moreover, it is still controversial whether the degree of cervical sagittal balance damage after LAMP is associated with the preoperative sagittal parameters [11, 23].

In our study, whether it is before or after surgery, patients in the LS group had larger T1s and CL, and smaller cSVA than those in the MS and HS group, which is consistent with previous reports that higher T1s tend to be accompanied by higher CA [8, 11]. Simultaneously, patients in the LS group also performed more sagittal alignment changes after LAMP, such as LOCL and increased cSVA, which suggests that smaller SCA requires more cervical curvature to supplement more energy expenditure and predict more changes after LAMP. Previous studies have shown that patients who had higher T1s could be accompanied by higher CL and greater effort to maintain cervical alignment balance [11, 23]. Our research results seem to apply this hypothetical conclusion to SCA as well, and it can be generalized to cervical myelopathy. It may explain that the patients with lower SCA are more susceptible to LAMP in sagittal balance. Although the incidence of postoperative kyphosis failed to reach significance, LOCL more than 5° or 10° appeared more regularly in the lower SCA group. The incidence of postoperative kyphosis in the LS group and the HS group was similar, which was about three times as much as that in the MS group. Therefore, we speculated that SCA might also be associated with sagittal alignment changes, so we established the multiple linear regression analysis model to try to correlate postoperative LOCL and preoperative measurements. It is unfortunate that only T1s was associated with LOCL in the multiple linear regression analysis, which is similar to the conclusion of previous research that T1s could be used as an appropriate predictor of postoperative cervical alignment change after LAMP [11]. Therefore, SCA does not seem to be a suitable predictor of LOCL. Interestingly, when we performed univariate linear regression analysis on SCA and LCL in the different subgroups, we found that no matter if SCA is too large or too small, there is a negative correlation with LOCL. When SCA changes within an appropriate range, it loses its relevance to LOCL. This indicates that SCA affects the change of cervical curvature to a certain extent. It may be due to the fact that smaller SCA requires more posterior cervical strength to maintain more cervical lordosis to ensure that the head is in a balanced state, the strength of the posterior cervical muscles in patients with too small SCA is reduced after LAMP and they are unable to maintain horizontal vision as before. In this case of decompensation, patients with too small SCA were more likely to lose cervical curvature under the change of head and cervical overall gravity. For patients with too high SCA, although they do not need too much curvature and muscle strength consumption to maintain horizontal vision, but excessive SCA is often accompanied by the forward movement of head and neck load-bearing axis, thus aggravating kyphosis and causing cervical imbalance. Most spinous processes, interspinous ligaments and supraspinous ligaments were removed after LAMP, which weakened the function of tether like stretching structure of posterior cervical ligament complex. The function of the posterior column to share the load transfer of the cervical spine is partly lost, which also results in the kyphotic alignment change. When the SCA fluctuates in a suitable range, it is easier to compensate for the changes of cervical sagittal alignment. Patients with middle SCA are not accompanied by large kyphosis force from the head, and the neck muscles do not need more physical labor to maintain horizontal vision and minimize the energy consumption related to the head weight. Therefore, the preoperative SCA value provides a reference for the postoperative cervical alignment changes. But actually after LAMP, cervical alignment changes can also be associated with factors such as the degree of cervical stiffness, bone mass, posterior muscle status, and disc degeneration. Therefore we had to consider the above factors if we wanted to explore the relationship of SCA with LOCL in depth.

Our study has several significant limitations. The first is related to the retrospective design. Moreover, the average follow-up time was too short, as well as relatively small sample sizes. Secondly, there is no comprehensive evaluation of postoperative clinical results, such as the Neck Disability Index (NDI), Japanese Orthopaedic Association (JOA) and the 36-Item Short-Form Health Survey (SF-36). According to our previous research [24], we found that a higher SCA was associated with worse quality of life. Perhaps this is because the increase of SCA is accompanied by the decrease of T1s and the loss of lordosis after LAMP, which would interfere with horizontal vision. Thus, patients may try to compensate cervical balance state by lowering T1s, resulting in the stretching of various muscles attached to the neck, which will trigger the threshold of pain and aggrandize energy consumption. So laminoplasty could be a good choice for patients with lower SCA. However, the relationship between too large or too small SCA and clinical efficacy score is still unknown, which has given us a lot of inspiration and may be the future research direction. Third, we did not collect the sagittal X-ray examination of the global spine, so the relationship between SCA and the global spine radiograph was not able to be further determined.

## Conclusion

Patients with lower SCA had more CL preoperatively and performed more LOCL after LAMP at 2 years of follow-up. Both too high or low preoperative SCA were negatively correlated with the degree of LOCL, while when the SCA fluctuates in a suitable range, it is easier to compensate for the changes of cervical sagittal alignment.

## Data Availability

The datasets generated and analysed during the current study are available from the corresponding author on reasonable request.

## Abbreviations

LOCL: Loss of cervical lordosis
LAMP: Laminoplasty
CSM: Cervical spondylotic myelopathy
OPLL: Ossification of posterior longitudinal ligament
SCA: Spino cranial angle
T1s: T1-slope
CL: C2-7 lordosis
cSVA: C2–C7 sagittal vertical axis
JOA: Japanese orthopaedic association
NDI: Neck disability index
SF-36: 36-Item short-form
